# Acute Alcoholism

**Published:** 1904-12

**Authors:** C. C. Stockard

**Affiliations:** Atlanta, Ga.


### ACUTE ALCOHOLISM.*

By C. C. STOCKARD, M.D.,

ATLANTA, GA.

Acute alcoholism in certain stages is a condition with which all of us are, by observation, only too familiar, and, it is not altogether unlikely that, in a company of scientific physicians even the size of this, there may be some who have had closer personal experience than that gained by observing others. In this I hope I am mistaken, for no class needs clear heads and steady hands more imperatively than we who have the lives of our brothers in charge.

Acute alcoholism varies in its symptoms according to the amount taken and the capacity of the individual, from vasomotor nerve paralysis resulting in flush of the cheeks, brightening of the eyes, and increased flow of ideas with volubility of tongue, through the usual symptoms of a drunk as seen on the street, viz.: inability to coordinate well, resulting from interference with cerebellum spinal cord action, rambling talk, which may be good humored and ridiculous, or violent and dangerous, according to the man's bent, and, finally to paralysis of the centers of sensation and motion, putting one in the condition commonly known as dead drunk, in which the temperature is lowered, and other evidences of vital depression are present, which may increase, if alcoholic absorption continues, to paralysis of the respiratory and circulatory centers, resulting in death.

*Read before the Atlanta Society of Medicine Nov. 17, 1904.

The first symptoms produced have long been construed to mean stimulation, but the best authorities are coming more and more to the belief that alcohol is never a stimulant but always the reverse. In a very able article recently read by Dr. Rutherford, of Bolton, England, he claims that "Alcohol, in the smallest dose, even, is a protoplasmic poison, and not a stimulant, impairing motion, nutrition, and reproduction, where it does not destroy; that the blood is poisoned and not fortified; the white and red cells, the oxygen carriers, the vitalizers, the sanitary police, and scavengers of the body, impaired, and that, instead of being a stimulant as has long been held, it is the reverse."

The diagnosis of acute alcoholism as commonly seen is easily recognizable even at quite a distance. When, however, as often happens, one is found in a comatose condition, with no knowledge of immediately preceding circumstances, it is often extremely difficult to make out whether or not alcohol is the cause, and if it can be proven that the patient has been drinking, it may be still that he has fallen and done injury to the head, or that some one may have struck him with the same result, and this too, without any visible evidence on the scalp being present. Under these conditions we may have acute alcoholism complicated with a meningeal hemorrhage, or a concussion of the brain. In such cases it is simply impossible to tell how much may be due to alcohol and how much to other causes, so that the safe thing to do is to empty the stomach, by emetic, or, preferably, the tube, afterward keeping the patient warm, and, if indicated, giving such stimulants as strychnine, digitalis, ammonia, and caffeine. These will be needed if the condition is due to alcohol.

Delirium tremens, being itself an acute condition, and, resulting from the continuous saturation of the system with alcohol for several days, or possibly a few weeks, comes, properly I presume, under the portion of the subject for tonight assigned to me. It is usually promptly relieved—at least within a few days—after stopping entirely the use of alcohol, being very different from the insanities resulting from chronic alcoholism, and lasting weeks, months, or even for years. In delirium tremens after one has been drinking excessively for some time, he begins to get nervous, apprehensive and even badly frightened. At first he sees unusual things, such as animals of various kinds making faces at him, or reptiles, insects, etc., with the eyes closed only, but later with the eyes open also he sees the most frightful objects. Hallucinations of hearing also appear and he distinctly hears voices, which he readily recognizes, planning and plotting all sorts of things against him. These hallucinations are always worse, from about five to ten P.M., and during the remainder of the twenty-four hours give little trouble. There is more or less tremor present in all cases.

Delirium tremens resembles quite closely in some of its characteristics the effects of cocaine indulgence, and also bromidism, and must be differentiated from them or the very opposite treatment from that needed may be given.

A case was recently sent to the Halcyon-Retreat from a neighboring city—the physicians pronouncing the case delirium tremens—and they were giving some whisky, fearing to stop too soon, and also a mixture containing bromide. I promptly recognized the condition as one of bromidism. The whisky and bromide were stopped at once, and in about two weeks the mental condition cleared up, when the gentleman stated that while suffering from nervousness he had bought four ounces of bromide sodium, and putting it in one-half pint of water, had taken a swallow when needed to relieve his nervousness. He declared he had not been drinking at all to excess.

Hyoscine sometimes produces a condition quite similar to delirium tremens also—but this passes away in a few hours. Knowing that the patient has been drinking heavily, and eliminating these other causes, delirium tremens is readily diagnosed. The treatment in these cases consists in the stoppage of alcohol with the administration of bromides, digitalis and strychnia, with such adjuvants as seem indicated. It is always necessary to give some hypnotic for a few nights, and for this purpose I know of nothing so prompt in its action as apomorphia, in doses of one-thirtieth of a grain, or even one-fortieth. The effect, however, is short, lasting usually about four hours, so that I sometimes give at the same time thirty grains of trional, which will usually secure a good night's sleep. When the bromide and digitalis have been kept up in sufficient doses for two or three days, the hypnotic will not be needed longer.

The necessity of having these patients carefully watched can not be too strongly emphasized, as, if left to themselves, in their thoroughly demoralized and frightened condition, there is no telling what may happen. A physician friend of mine had one to run seven miles, without any clothing on, and in freezing weather, in consequence of which he died.

